# Durable Corrosion Resistance of Copper Due to Multi-Layer Graphene

**DOI:** 10.3390/ma10101112

**Published:** 2017-09-21

**Authors:** Abhishek Tiwari, R. K. Singh Raman

**Affiliations:** 1 Department of Mechanical and Aerospace Engineering, Monash University, Clayton, VIC 3800, Australia; raman.singh@monash.edu; 2 Department of Mechanical Engineering, Bansal Institute of Research and Technology, Kokta, Anand Nagar, Bhopal, Madhya Pradesh 462021, India; 3 Department of Chemical Engineering, Monash University, Clayton, VIC 3800, Australia

**Keywords:** copper, graphene, EIS, Raman spectroscopy, chemical vapour deposition, corrosion

## Abstract

Ultra-thin graphene coating has been reported to provide considerable resistance against corrosion during short-term exposures, however, there is great variability in the corrosion resistance due to graphene coating in different studies. It may be possible to overcome the problem of hampered corrosion protection ability of graphene that is caused due to defective single layer graphene by applying multilayer graphene. Systematic electrochemical characterization showed that the multilayer graphene coating developed in the study provided significant corrosion resistance in a chloride solution and the corrosion resistance was sustained for long durations (~400 h), which is attributed to the multilayer graphene.

## 1. Introduction

Corrosion and its mitigation is expensive, as it costs ~4% of GDP of any developed economy. Since corrosion is an age-old problem, several mitigation strategies have been investigated and adopted with different degrees of success. The use of graphene as an ultra-thin coating has emerged as a novel and exciting approach to the corrosion protection [1,2,3,4,5,6,7,8,9,10,11,12,13,14,15,16]. The remarkable properties of graphene include its very high charge carrier mobility [17,18], high optical transparency (97.7%) [19], flexibility (spring constant 1–5 N/m) [20], a breaking strength 100 times greater than the strongest steel with a tensile modulus (stiffness) of 1 TPa [21], scratch resistance [22], record electrical conductivity [23], the highest current density (million times that of Cu) [24] and thermal conductivity of (4.84 ± 0.44) × 10^3^ to (5.30 ± 0.48) × 10^3^ Wm^−1^ K^−1^ (outperforming diamond) [25], impermeability even to the smallest atomic size gas (i.e., He) [26], and chemical inertness [27]. The specific properties that make graphene an excellent candidate for corrosion resistant barrier coating are its remarkable chemical inertness, impermeability for fluids, and high toughness. An ultra-thin graphene coating has been shown to improve corrosion resistance of copper by two orders of magnitude in an aggressive solution simulating to sea-water [1]. However, graphene coatings on Cu investigated by different research groups have shown varying degrees of corrosion resistance—from insignificant to two orders of magnitude [1,2,3]. In fact, recently, some researchers have reported graphene coated copper to corrode at considerably greater rates than uncoated copper. The considerable variations in corrosion resistance of chemical vapour deposition (CVD) graphene coated copper arise due to the lack of complete surface coverage, irregularities, presence of defects, cracks, domain boundaries, wrinkles, and folds (that are inherent in CVD graphene) [28]. In a study, the oxidation resistance of iron and copper foils was achieved by coating them with reduced graphene oxide (rG-O) multilayers when subjected to heat treatment at 200 °C in air for 2 h [29].

**Figure 1:**
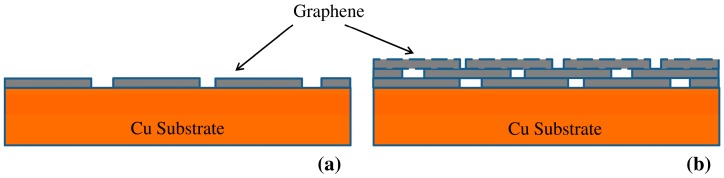
Schematics of: (**a**) single layer graphene with bare locations at the graphene domain boundaries; (**b**) multilayer graphene showing the graphene domain boundaries of inner layer to be masked by the immediate upper layer.

In the light of the recent research findings on graphene for corrosion resistance [1,2,3,4,5,28,30], it may be necessary to have a full surface coverage of graphene for a durable corrosion resistance. In this study, we first hypothesize that a more effective surface coverage and corrosion resistance may be attained due to multilayer graphene (instead of single layer graphene) as schematically shown in Figure 1. We validated this hypothesis by developing multi-layer graphene on copper, and then experimentally demonstrated the multilayer graphene coating to provide considerably durable corrosion resistance. 

## 2. Experimental Methods

### 2.1. Deposition of Graphene Coating on Copper

The copper foils of thickness 0.05 mm were procured from Storm Copper Components, Decatur, TN, USA. The copper samples were plasma cleaned under Ar/O_2_ for 2 min using Solarus Advanced Plasma System (Model 950, Gatan, Inc., Pleasanton, CA, USA) and cut to the size of 1 × 1 cm and placed in the hot zone of quartz tube of a furnace for chemical vapour deposition (CVD) of graphene. The CVD set-up, shown in Supplementary Section S1 (Figure S1), was designed and built for graphene synthesis using n-hexane as the hydrocarbon source, a tubular furnace capable of operating at temperatures up to 1600 °C, a vacuum pump, and mass flow controllers for n-hexane and Ar/H_2_ mixture.

The quartz tube was first pumped down to a pressure of 70 mTorr, and then a gas mixture of 85% Ar and 15% H_2_ was introduced at a pressure of 6 to 9 Torr and flow rate of 400 sccm, to create a reducing atmosphere to avoid oxidation of copper samples at high temperatures. The furnace is heated to 1000 °C while maintaining a reducing atmosphere of Ar/H_2_ flow. The copper coupons were annealed for 50 min at 1000 °C under the reducing atmosphere, before Ar/H_2_ flow was replaced with n-hexane vapour that was passed at 2 sccm and 500 mTorr for 7–10 min. Then, the copper coupons were cooled under the flow of Ar/H_2_ at 400 sccm and at a pressure of 8 to 9 Torr.

### 2.2. Characterization of Graphene

The coating developed as discussed earlier was characterized using Raman spectroscopy, optical microscopy, and scanning electron microscopy. 

Raman spectroscopy provides confirmatory information on whether the deposited layer is graphene. The deposited layer was characterized using Renishaw micro-Raman Spectrometer (Model inVia, Renishaw plc, Wotton-under-Edge, Gloucestershire, UK) HeNe (632.8 nm) laser operating at 100% power. Extended scans (10 s) were performed for a spectral range between the Raman shifts of 150 to 3200 cm^−1^ with a laser spot size of 1 μm. 

The optical micrographs of bare copper and graphene coated copper were obtained using Olympus BX 51 (Model - BX51TRF, Olympus Corporation, Tokyo, Japan) optical microscope. 

The scanning electron microscopy (SEM) images of graphene coated copper samples were obtained using JEOL 7001 with an accelerating voltage of 10 kV at 12 nA probe current and 10 mm working distance. 

### 2.3. Corrosion Resistance Due to Graphene Layer 

Electrochemical impedance spectroscopy (EIS) was performed on the graphene coated and bare copper samples in 0.1 M NaCl solution using a Princeton Applied Research (PAR) potentiostat (Model 2273, Princeton Applied Research, Advanced Measurement Technology, Inc., Oak Ridge, TN, USA) and an electrochemical cell with three electrodes (specimens with an exposed area of 0.785 cm^2^ acted as the working electrode, platinum mesh as counter electrode and saturated calomel electrode as the reference electrode). E_corr_ vs. time plots were generated after immersion in 0.1 M NaCl for 1 h, to ascertain the stabilized open circuit potential (OCP). Since the perturbation potential for the EIS test was 10 mV, any fluctuation in the open circuit potential of less than 10 mV for a period of 1000 s was considered as a stable potential for the purpose of EIS experiments. A small sinusoidal potential of 1 to 10 mV AC was applied which rendered the system pseudo-linear. EIS runs were carried out by applying a sinusoidal signal at E_corr_ with perturbation potential of 10 mV. Impedance response was measured over frequencies between 1 MHz and 10 mHz, recording 10 points per decade of frequency using Powersuit software (version 2.58). These frequencies are chosen such that they reach asymptotic limits in which the imaginary impedance tends to zero at the lowest and highest frequencies of the employed frequency range. 

Impedance analysis was carried out using PAR ZSimpWin package for Windows for frequencies from 10,000 Hz to 0.1 Hz which prevented misinterpretation of any artefacts that may be present in the high frequency region or the scatter in the low frequency region.

## 3. Results

### 3.1. Characterization of Graphene on Copper

**Figure 2:**
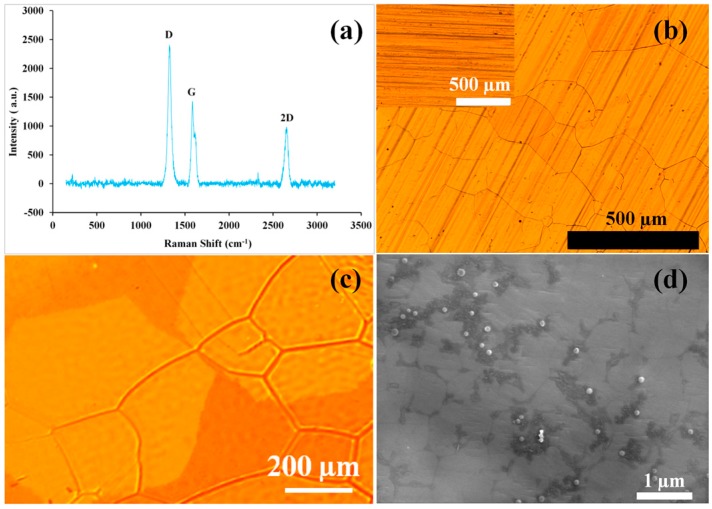
Graphene coated copper (**a**) Raman spectrum; (**b**) optical micrograph of graphene (inset, bare copper) in this study; (**c**) optical micrograph (from [35]) showing copper grain boundaries underneath the hexagonal graphene grains (Reprinted with permission from T. Wu, G. Ding, H. Shen, H. Wang, L. Sun, D. Jiang, X. Xie, and M. Jiang, Advanced Functional Materials 23 198–203 (2013). Copyright 2013 John Wiley and Sons); and (**d**) scanning electron micrograph.

The two Raman signature peaks are G peak at 1580 cm^−1^ and 2D peak at 2700 cm^−1^ (Ferrari et al. [31]). The ratio of intensities of G and 2D peaks (I_G_/I_2D_), i.e., a measure of the number of graphene layers, is ~0.36 for a single layer, and >1 for multi-layer graphene [32,33]. The I_G_/I_2D_ ratio in a typical Raman spectrum in Figure 2a (i.e., a representative of 5–6 such spectra (see Supplementary Section S2, Figures S2 and S3 for reproducibility)) of the graphene films deposited in the present study is 1.42, which indicates 4–5 layers of graphene. The D-peak at 1350 cm^−1^ signifies the extent of defects in graphene [34]. The high intensity of D peak in this study indicates that the graphene layers had considerable defects, such as vacancies and strained hexagonal/non-hexagonal (pentagon or heptagon) distortions that result in corrugation and twisting of layers [34].

Graphene coated copper has features of thermally etched grain boundaries (Figure 2b) which is similar to the reported feature shown in Figure 2c [35]. Figure 2b shows that even 4–5 layers of graphene are optically transparent. Unlike those in Figure 2c, no isolated hexagonal graphene grains are seen in Figure 2b, suggesting the copper surface is fully covered with graphene in this study.

Scanning electron microscopy (SEM) of graphene coated Cu in the present study shows dark areas in Figure 2d that have been attributed either to graphene flakes or to wrinkles [36]. The thermal expansion coefficient of graphene is negative [37] and that of copper is positive. Therefore, on cooling from the graphene deposition temperature in the CVD process, graphene expands while copper shrinks, leading to wrinkles of graphene. 

### 3.2. Electrochemical Impedance Spectroscopy (EIS) for Characterization of Corrosion Resistance

**Figure 3:**
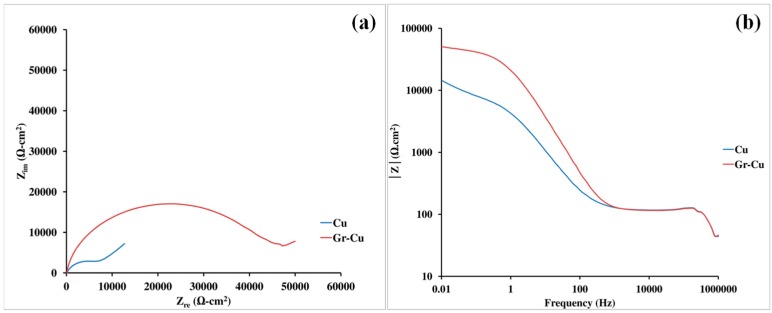
Corrosion resistance of bare copper (Cu), and graphene coated copper (Gr-Cu) in 0.1 M NaCl: (**a**) Nyquist plots (bare copper data reprinted with permission from [38], R. K. Singh Raman, A. Tiwari, JOM, 66, (4), 637-642 (2014). Copyright 2014 Springer) and (**b**) Bode modulus plots (bare copper data reprinted with permission from [39], A. Tiwari, R. K. Singh Raman, Corrosion & Prevention 2013 Conference, Brisbane, Australia, 2013; pp 1-7. Copyright 2013 Australasian Corrosion Association Inc.).

The diameter of semicircular loop of a Nyquist plot and the magnitude of impedance at the lowest frequency in a Bode impedance plot are broad measures of corrosion resistance. The Bode and Nyquist plots of graphene coated and uncoated samples show the impedance of graphene coated copper to be 4–5 times higher than the uncoated copper (Figure 3). The improvement in corrosion resistance due to graphene coating is attributed to the multilayer graphene. Bode phase plots have been discussed in Supplementary Section S3 (Supplementary Figure S4). 

**Figure 4:**
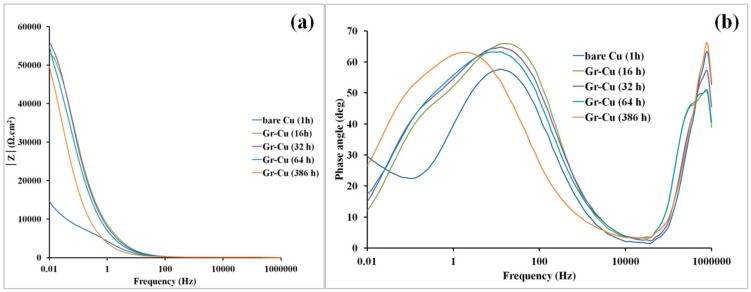
(**a**) Bode modulus and (**b**) Bode phase plots for bare and graphene coated copper (Gr-Cu) after immersion for different durations (16–386 h) in 0.1 M NaCl (Note: bare Cu was immersed only for 1 h).

Though the corrosion resistance due to the multilayer graphene is not quite impressive (only 4–5 times), it is still of great interest to assess whether this corrosion resistance was durable, and hence, EIS study was undertaken after immersion in 0.1 M NaCl for different durations (1, 16, 32, 64, and 386 h). The EIS data representing the improvement in corrosion resistance are reproducible as established in Supplementary Section S4 (see Figure S5). A consolidated set of Bode impedance plots (Figure 4a) shows the impedance (corrosion resistance) of graphene coated specimen at the lowest frequency to be ~5 times greater than that for the pure copper even after a long immersion for 386 h. For comparison, the spectra for bare copper after 1 h of immersion is also presented. The broad nature of phase angle peaks indicates two time constants in each case (Figure 4b). The two time constants are attributed to the presence of two interfaces, i.e., the graphene coating/solution interface and the metal/solution interface in the case of graphene coated copper whereas the corrosion products/solution interface and metal/solution interface in the case of bare copper [1]. The surface of the pure copper will quickly develop a layer of copper oxide/hydroxide in the corrosive solution. Quantitative determination of characteristic parameters of surface layer (graphene or copper oxide/hydroxide)—such as capacitance, resistance, etc.—by simulation of the experimental EIS data using an appropriate equivalent electrical circuit (EEC) based on the established corrosion mechanism is described in the Supplementary Section S5 (see Figure S6). 

The data in Supplementary Table S1 suggest that, for the multilayer graphene coated Cu, the sum of pore resistance and the resistance offered by the interface of multilayer graphene and electrolyte interface (R_c_) values, that are the measure of the corrosion resistance are 4.718 × 10^4^ Ωcm^2^, 4.52 × 10^4^ Ωcm^2^, 5.249 × 10^4^ Ωcm^2^, 4.875 × 10^4^ Ωcm^2^, and 6.260 × 10^4^ Ωcm^2^ after the immersions for 1, 16, 32, 64, and 386 h respectively, whereas that of the uncoated/bare copper after 1 h immersion is 1.245 × 10^4^ Ωcm^2^ (for bare copper, it is the sum of pore resistance and the resistance offered by metal/electrolyte interface). Therefore, the data infer the multilayer graphene coating to improve, corrosion resistance of copper by ~5 times, and more importantly sustains this improvement for 386 h, which is largely consistent with the data in Figure 3 and Figure 4. The lower Q_f_ of multilayer graphene which suggests a lower number of conductive pathways in multilayer graphene film whereas the relatively low value of C_dl_ of multilayer graphene film indicates lower exposure of metal–electrolyte interface (see section S5 supplementary material for definition of Q_f_ and C_dl_). The exponent of CPE is very close to 1, indicating a fully capacitative nature of coating. The low Chi squared value represents fair accuracy of the results. Supplementary Figure S7a–c show the fitting of simulated data with experimentally observed data for graphene coated copper after 1, 64, and 386 h of immersions, respectively. As evident from Supplementary Figure S7a–c, the experimental plots match well with the simulated data in the frequency region 0.1 to 10,000 Hz. The total error in impedance measurements in the simulation of the experimental data, when using the EEC shown in Supplementary Figure S6 was less than 4% in all the experiments. The associated Chi squared values were also relatively low. The error and Chi squared data confirms the validity of the employed EEC (Supplementary Figure S6) as well as the degradation mechanism. 

## 4. Discussion

It is noted that graphene in the study of Singh Raman et al. [1] had a smaller D peak in Raman spectrum as compared to prominent D peak for the graphene developed in this study (Figure 2). Therefore, lesser improvement in corrosion resistance due to graphene coating in this study as compared to that in the study of Singh Raman et al. [1] (as evident by the EIS data in Figure 4 and those reported in [1]) can be attributed to the difference in nature of graphene coating in the two studies. The stronger D peak (defect peak) in Figure 2 indicates more conducting pathways for electrolytes through these defects. 

Contrary to the observations of improvement in the corrosion resistance due to graphene coating in this study and those reported in the literature [1,2,3], a very recent study by Schriver et al. [4] has reported the longer-term oxidation/corrosion resistance of graphene-coated copper in air at 250 °C to be considerably inferior to the uncoated copper. However, the researchers have also provided evidence of considerable defects in their graphene coating. Such defects were evidently the locations where oxidation started whereas the areas where the coating remained intact continued to show excellent oxidation/corrosion resistance for the durations of the experiment. In fact, prior to this recent report [4], Singh Raman et al. [1] had clearly suggested that, in the case of the coatings that do not provide complete surface coverage, the corrosion resistance could be inferior, because of the highly cathodic nature of graphene/graphite. Graphene coating providing varying degrees of corrosion resistance and the extent of defect contents to be the cause of this variability is evident from comparison of results of different groups [1,2,3]. Zhou et al. [30] have also suggested enhanced corrosion of copper in the presence of graphene during long exposures (six months). In their study, Raman spectroscopy confirmed the presence of a single layer graphene on copper, however, the surface coverage of graphene was questionable. 

The above discussion would suggest that graphene domain boundaries may be the major defect type that may impair corrosion/oxidation resistance. This assumption is consistent with the results and schematics for deterioration in corrosion resistance of graphene coated copper reported in the recent studies [4,30]. Therefore, an effective way to improve corrosion resistance may be either to develop as much of a defect free graphene coating as possible (as was achieved in study by Singh Raman et al. [1]) or to plug/cover the defect areas (domain boundaries). It is suggested that the development of the multilayer graphene can achieve the latter, as schematically shown in Figure 1. 

The role of plugging/masking the graphene domain boundary locations in effectively improving corrosion resistance is strongly corroborated by the findings of Hseih et al. [40]. In this painstaking work, the researchers plugged the graphene domain boundaries by atomic layer deposition (ALD) of aluminium oxide at such locations. The graphene layer with this ALD aluminium oxide provided remarkable resistance/passivation against corrosion. 

In the light of the above discussion, the superior surface coverage of the multilayer graphene led to a durable corrosion resistance for the entire test duration (~400 h) in the present study. 

In the context of the beneficial effect of the multilayer graphene in corrosion resistance, the work of Prasai et al. [2] that compared the corrosion resistance offered by CVD graphene and mechanically transferred graphene on Ni is interesting. They demonstrated that CVD grown graphene improved corrosion resistance of Ni by 20 times, whereas mechanically transferred graphene (two or four layers) provided a maximum improvement of only four times (Figure S8). This is because the CVD graphene on Ni is likely to provide multilayer graphene as confirmed by Raman spectroscopy, and therefore, an inherent likelihood of good corrosion resistance on the basis of the mechanism similar to one shown in Figure 1b. On the other hand, the multilayer graphene developed by mechanical transfer will invariably leave channels for transport of corrosive ions and hence inferior corrosion resistance for mechanically transferred graphene layer on Ni in comparison to the CVD graphene on Ni (Figure S8). 

It may be relevant to note that the multilayer CVD graphene on Ni may be more defect-free because the mechanisms of deposition of graphene on Ni and Cu are different. Graphene deposition on the metals having considerable solubilities of carbon (e.g., Ni) progresses by an intricate mechanism. In this mechanism, the carbon produced due to hydrocarbon decomposition at the metal surface diffuses into the bulk metal, and is subsequently rejected out during cooling since carbon solubility decreases considerably with temperature. On the other hand, metals having little/very limited solubility of carbon at reaction temperature—e.g., Cu—are more likely to develop a single layer graphene instead of multilayer graphene because of the catalytic effect is likely to cease after the first layer is deposited [41]. However, it is not uncommon to find multilayer graphene on copper [7,42,43,44,45,46], as also observed in the present study.

## 5. Conclusions

In conclusion, graphene coating was synthesized on copper using a chemical vapour deposition technique. Electrochemical results indicated that the graphene coating acted as a corrosion barrier for copper and provided a durable corrosion protection in 0.1 M NaCl. Contradictory findings as reported in the literature regarding corrosion protection ability of graphene are attributed to the quality of graphene. Corrosion protection ability of graphene can be retained for long exposure times by applying multilayer graphene instead of single layer graphene. Multilayer graphene is suggested to be effective in blocking the pathways of corrosive species.

## Supplementary Materials

The following are available online at www.mdpi.com/1996-1944/10/10/1112/s1, Figure S1. Experimental set-up for CVD graphene synthesis; Figure S2. Optical micrograph and Raman mapping for graphene coated copper sample; Figure S3. Raman spectrum of graphene coated Cu on different spots showing uniformity of multilayer graphene over the sample surface; Figure S4. Bode phase angle plots for Cu and graphene coated copper (Gr-Cu) sample; Figure S5. Bode impedance plots for graphene coated copper after immersion in 0.1 M NaCl for (a) 1 h (Gr-Cu (1h)-sample-2 data reprinted with permission from A. Tiwari, R. K. Singh Raman, Corrosion & Prevention 2013 Conference, Brisbane, Australia, 2013; pp 1–7. Copyright 2013 Australasian Corrosion Association Inc.), (b) 64 h, and (c) 386 h of immersion; Figure S6. Equivalent electrical circuit (EEC) for corrosion of bare and graphene coated copper; Figure S7. Curve fitting of experimental and simulated Bode plots for graphene coated copper after immersion in 0.1 M NaCl for (a) 1 h, (b) 64 h, and (c) 386 h of immersion; Figure S8. Corrosion rate: Gr/Ni is graphene coating on Ni by CVD, tr2Gr/Ni, and tr4Gr/Ni represent mechanically transferred 2 and 4 layers onto Ni, and Ni is uncoated-Ni (Reprinted with permission from D. Prasai, J. C. Tuberquia, R. R. Harl, G. K. Jennings, and K. I. Bolotin, ACS Nano 6 (2), 1102-1108 (2012)., Copyright 2012 American Chemical Society.); Table S1. Quantitative analysis of EIS data by modelling using model EEC: R_s_(Q_f_[R_f_(C_dl_R_c_)]).
