# Supplementary material for: Durable Corrosion Resistance of Copper Due to Multi-Layer Graphene
Source: Materials (Basel). 2017 Sep 21;10(10):1112. doi: 10.3390/ma10101112 (PMC5666918; doi:10.3390/ma10101112)
Supplement: Supplementary file 1 [file materials-10-01112-s001.pdf]

Durable Corrosion Resistance of Copper due to Multi-Layer Graphene

Abhishek Tiwari [1,2,*] , *R. K. Singh Raman* [1,3]

[1]Department of Mechanical and Aerospace Engineering, Monash University, Clayton, VIC 3800, Australia

[2]Department of Mechanical Engineering, Bansal Institute of Research and Technology, Kokta, Anand Nagar, Bhopal, Madhya Pradesh 462021, India

[3]Department of Chemical Engineering, Monash University, Clayton, VIC 3800, Australia

[*] Author to whom correspondence should be addressed. *Electronic mail:* abhishektiwariiitr@gmail.com

Supplementary Material

S1. CVD reactor for graphene synthesis: The schematic diagram of experimental set-up for CVD graphene synthesis is as shown in Figure S1.

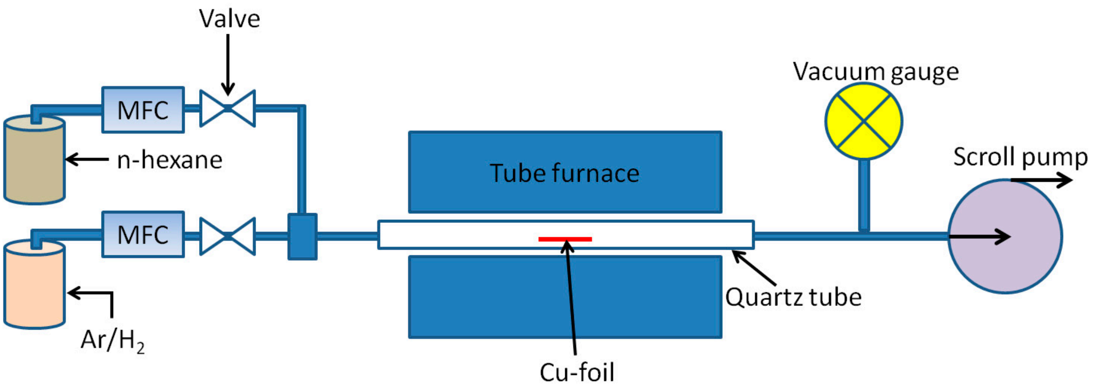

FIG. S1. Experimental set-up for CVD graphene synthesis.

S2. Uniformity of Graphene Coating: The uniformity of multilayer graphene was determined by Raman mapping over a large area of grapheme-coated copper sample, as shown in Figure S2. Though we have presented only five representative Raman spectra, hundreds of similar Raman spectra were obtained for Raman mapping confirming uniformity of multilayer graphene, as shown in Figure S3.

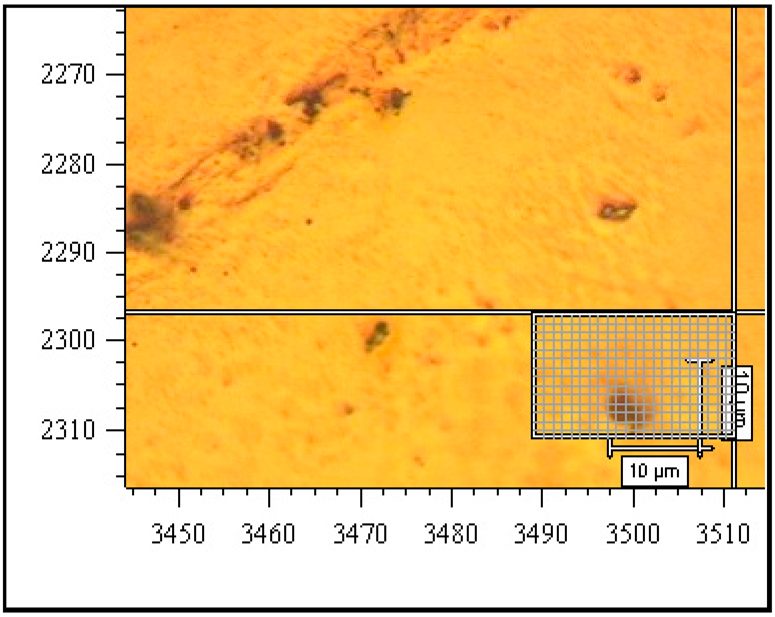

FIG. S2. Optical micrograph and Raman mapping for graphene coated copper sample.

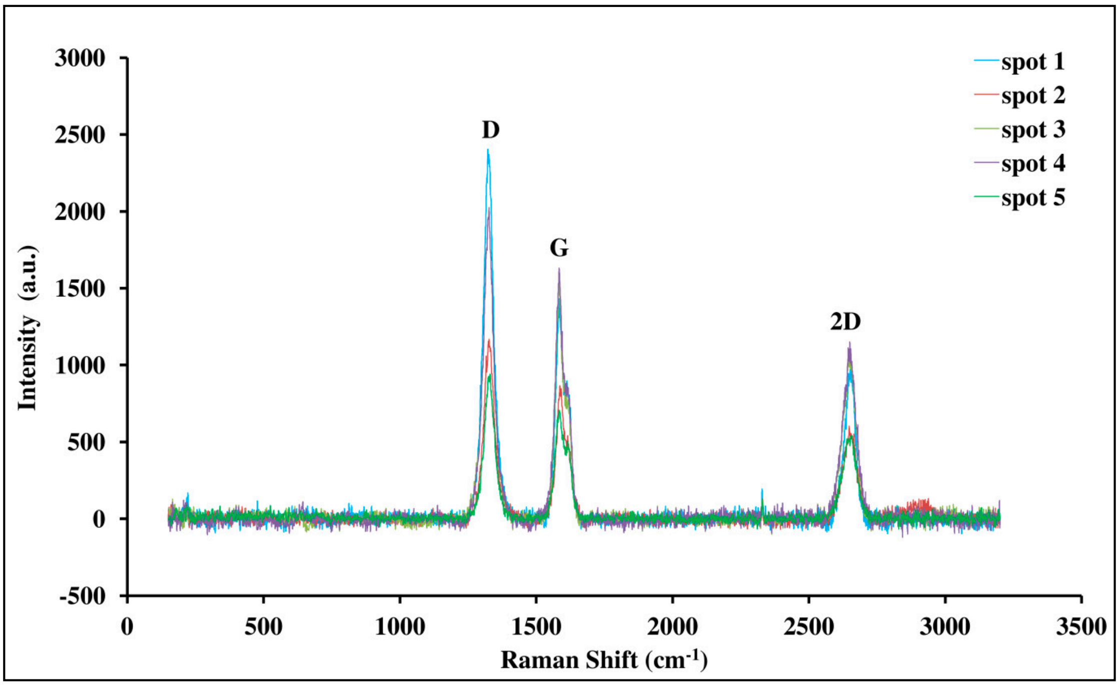

FIG. S3. Raman spectrum of graphene coated Cu on different spots showing uniformity of multilayer graphene over the sample surface.

S3. Bode phase angle plots: The Bode phase plots for graphene coated and uncoated Cu have been shown in Figure S4 for 1 h immersion in 0.1 M NaCl solution. The broad nature of

phase angle peaks in these Bode phase plots indicates two time constants in each case (Figure S4). The two time constants are attributed to the presence of two interfaces, i.e., the graphene coating/solution interface and the metal/solution interface in the case of graphene coated copper compared to the corrosion products/solution interface and metal/solution interface in the case of bare copper [1].

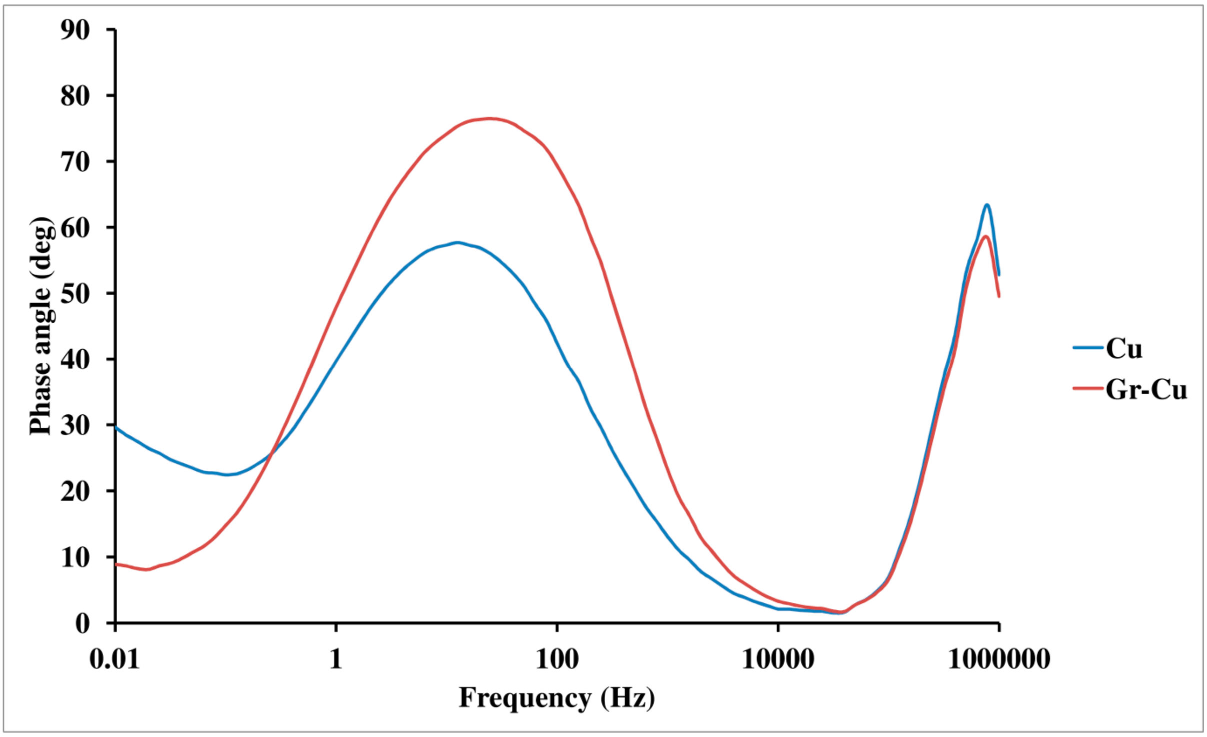

FIG. S4. Bode phase angle plots for Cu and graphene coated copper (Gr-Cu) sample.

S4. Reproducible Corrosion Resistance Data: Duplicate EIS runs were undertaken after immersion of graphene coated Cu for different durations (1, 16, 32, 64, and 386 h). Bode impedance plots for duplicate runs for immersions of 1, 64, and 386 h are represented in Figure S5.

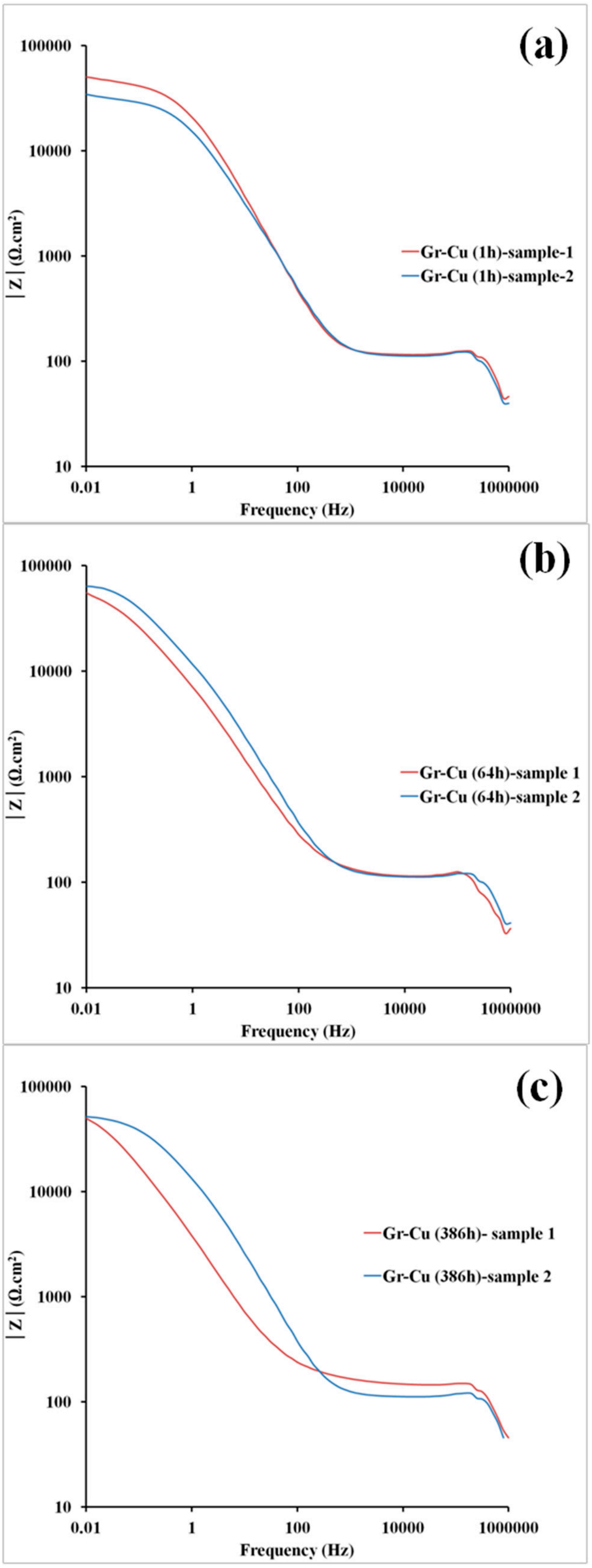

FIG. S5. Bode impedance plots for graphene coated copper after immersion in 0.1 M NaCl for (**a**) 1 h (Gr-Cu (1h)-sample-2 data reprinted with permission from [2], A. Tiwari, R. K. Singh Raman, Corrosion & Prevention 2013 Conference, Brisbane, Australia, 2013; pp 1-7. Copyright 2013 Australasian Corrosion Association Inc.), (**b**) 64 h, and (**c**) 386 h of immersion.

S5. Simulation of EIS Data: The three main criteria to be fulfilled while choosing an appropriate EEC for corrosion process are: (i) low Chi squared value, (ii) minimum total error and relative error in calculation of individual parameters, and (iii) consistency with a corrosion mechanism. An EEC with two time constants, as shown in Figure S6, was used to simulate the experimental impedance data.

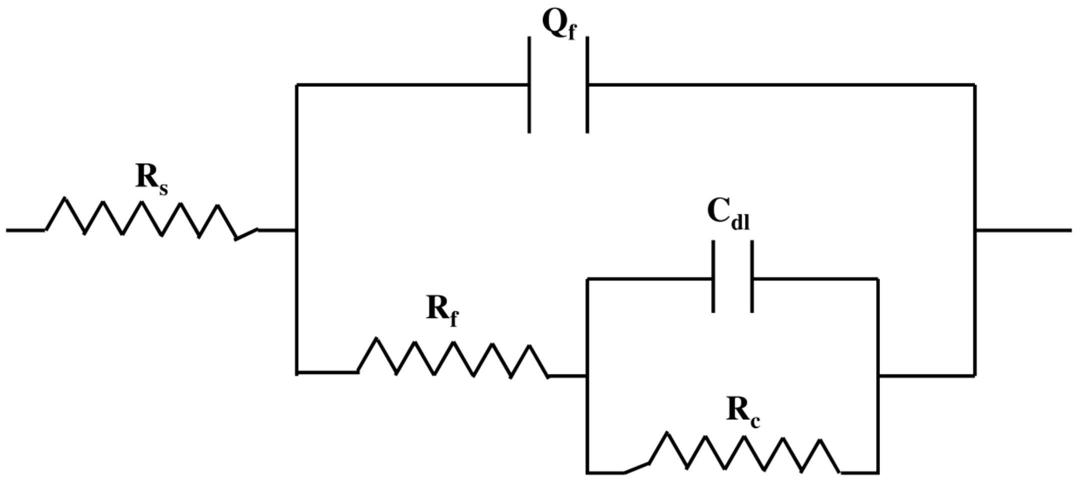

FIG. S6. Equivalent electrical circuit (EEC) for corrosion of bare and graphene coated copper.

The nested EEC was specifically chosen because of interconnected nature of the coating defects (such as graphene grain boundaries for the graphene coated Cu or the oxide/hydroxide porosity for the bare Cu). In this EEC, R_s is the solution resistance and the graphene coating is represented by a parallel combination of the constant phase element (CPE), Q_f, and the film resistance, R_f. The electrical double layer is represented by capacitance (C_{dl}) and a charge transfer resistance (R_c). In the case of graphene coated Cu, R_f denotes film resistance or pore resistance, and R_c denotes charge transfer resistance (i.e., resistance offered by the interface of multilayer graphene and electrolyte interface). In the case of bare Cu, R_f denotes film resistance or pore resistance and R_c denotes charge transfer resistance, i.e., resistance offered by metal/electrolyte interface.

In the simulation of the experimental data, the CPE behaviour is generally attributed to the distributed surface reactivity, roughness, and electrode porosity [3, 4]. Impedance analysis as carried out using PAR ZSimpWin package for Windows for frequencies between 10000 Hz to 0.1 Hz is shown in Table S1.

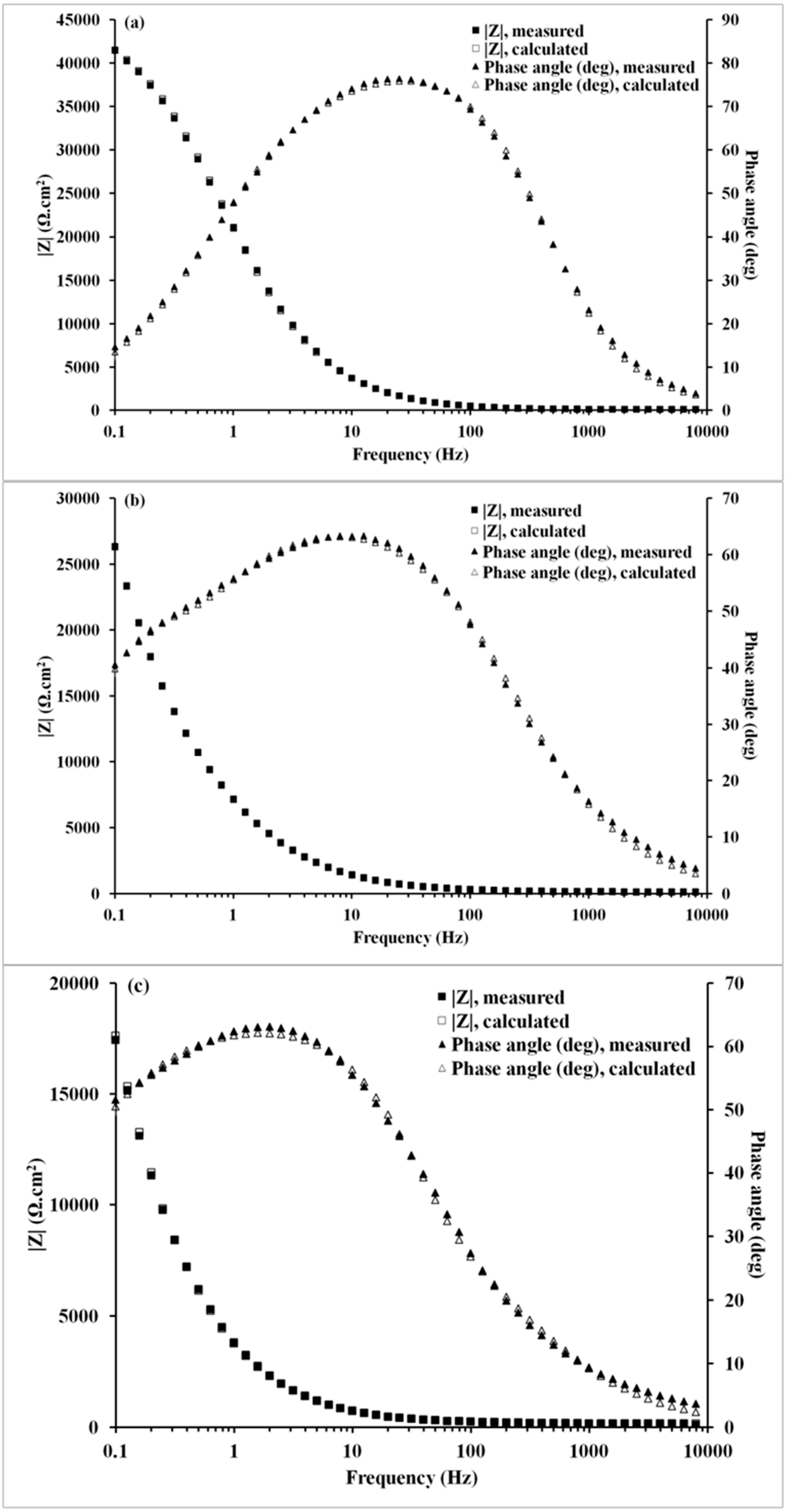

FIG. S7. Curve fitting of experimental and simulated Bode plots for graphene coated copper after immersion in 0.1 M NaCl for (**a**) 1 h, (**b**) 64 h, and (**c**) 386 h of immersion.

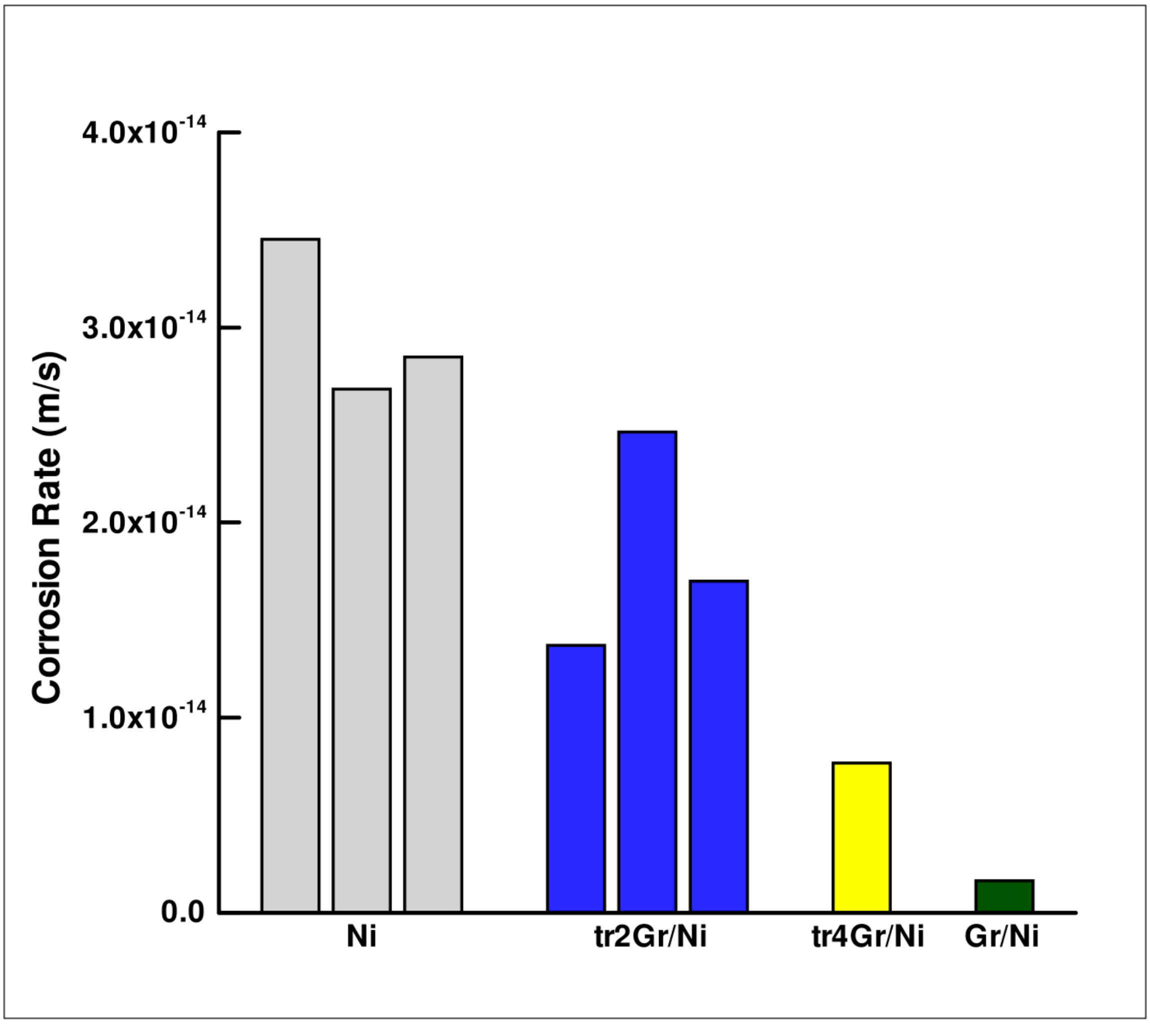

FIG. S8. Corrosion rate: Gr/Ni is graphene coating on Ni by CVD, tr2Gr/Ni, and tr4Gr/Ni represent mechanically transferred 2 and 4 layers onto Ni, and Ni is uncoated-Ni. [5] (Reprinted with permission from [5] D. Prasai, J. C. Tuberquia, R. R. Harl, G. K. Jennings, and K. I. Bolotin, ACS Nano 6 (2), 1102-1108 (2012)., Copyright 2012 American Chemical Society.)

Table S1. Quantitative analysis of EIS data by modelling using model EEC: $R_s(Q_f[R_f(C_{dl}R_c)])$

**Sample, immersion time**	**R_f (Ωcm^2)**	**R_c(Ωcm^2)**	**Q_f (F/cm^2)**	**n**	**C_{dl} (F/cm^2)**	**Chi squared value**	**Total error (%)**	**Corrosion Resistance (R_f+R_c) (Ωcm^2)**
Pure Cu, 1h	8.085×10^3	4.365×10^3	3.885×10^{-5}	0.7687	5.891×10^{-4}	8.533×10^{-5}	0.9238	1.245×10^4
Gr-Cu, 1h	15.92	4.716×10^4	6.079×10^{-6}	0.6569	2.875×10^{-6}	4.368×10^{-4}	2.09	4.718×10^4
Gr-Cu, 16h	1.818×10^4	2.702×10^4	1.783×10^{-5}	0.8291	3.666×10^{-5}	4.475×10^{-4}	2.115	4.52×10^4
Gr-Cu, 32h	2.384×10^4	2.865×10^4	2.305×10^{-5}	0.7968	4.08×10^{-5}	2.629×10^{-4}	1.621	5.249×10^4
Gr-Cu, 64h	2.542×10^4	2.333×10^4	2.849×10^{-5}	0.78	4.536×10^{-5}	3.104×10^{-4}	1.762	4.875×10^4
Gr-Cu, 386h	4.978×10^2	6.21×10^4	6.38×10^{-5}	0.7123	4.864×10^{-6}	3.314×10^{-4}	1.82	6.260×10^4

REFERENCES

1. Singh Raman, R. K.; Chakraborty Banerjee, P.; Lobo, D. E.; Gullapalli, H.; Sumandasa, M.; Kumar, A.; Choudhary, L.; Tkacz, R.; Ajayan, P. M.; Majumder, M., Protecting copper from electrochemical degradation by graphene coating. *Carbon* **2012,** 50, (11), 4040-4045.
2. Tiwari, A.; Singh Raman, R. K., Multilayer Graphene Coating on Copper for Corrosion Mitigation. In *paper #123 presented at the Corrosion & Prevention 2013 Conference*, Australasian Corrosion Association Inc.: Brisbane, Australia, 2013; pp 1-7.
3. Barsoukov, E.; Macdonald, J. R., *Impedence Spectroscopy: Theory, Experiment, and Applications*. Second ed. ed.; John Wiley & Sons, Inc.: Hobokon, New Jersey, 2005.
4. Chakraborty Banerjee, P.; Singh Raman, R. K., Electrochemical impedance spectroscopic investigation of the role of alkaline pre-treatment in corrosion resistance of a silane coating on magnesium alloy, ZE41. *Electrochimica Acta* **2011,** 56, (11), 3790-3798.
5. Prasai, D.; Tuberquia, J. C.; Harl, R. R.; Jennings, G. K.; Bolotin, K. I., Graphene: Corrosion-Inhibiting Coating. *ACS Nano* **2012,** 6, (2), 1102-1108.
